# Dynamics of regional lung aeration determined by electrical impedance tomography in patients with acute respiratory distress syndrome

**DOI:** 10.1186/2049-6958-7-44

**Published:** 2012-11-15

**Authors:** Sven Pulletz, Matthias Kott, Gunnar Elke, Dirk Schädler, Barbara Vogt, Norbert Weiler, Inéz Frerichs

**Affiliations:** 1 Department of Anaesthesiology and Intensive Care Medicine, Klinikum Osnabrück, Am Finkenhügel 1, 49076 Osnabrück, Germany; 2 Department of Anaesthesiology and Intensive Care Medicine, University Medical Center Schleswig-Holstein, 24105 Keil, Germany

**Keywords:** Acute lung injury, Electrical impedance tomography, EIT, Respiratory time constants

## Abstract

**Background:**

Lung tissue of patients with acute respiratory distress syndrome (ARDS) is heterogeneously damaged and prone to develop atelectasis. During inflation, atelectatic regions may exhibit alveolar recruitment accompanied by prolonged filling with air in contrast to regions with already open alveoli with a fast increase in regional aeration. During deflation, derecruitment of injured regions is possible with ongoing loss in regional aeration. The aim of our study was to assess the dynamics of regional lung aeration in mechanically ventilated patients with ARDS and its dependency on positive end-expiratory pressure (PEEP) using electrical impedance tomography (EIT).

**Methods:**

Twelve lung healthy and twenty ARDS patients were examined by EIT during sustained step increases in airway pressure from 0, 8 and 15 cm H_2_O to 35 cm H_2_O and during subsequent step decrease to the corresponding PEEP. Regional EIT waveforms in the ventral and dorsal lung regions were fitted to bi-exponential equations. Regional fast and slow respiratory time constants and the sizes of the fast and slow compartments were subsequently calculated.

**Results:**

ARDS patients exhibited significantly lower fast and slow time constants than the lung healthy patients in ventral and dorsal regions. The time constants were significantly affected by PEEP and differed between the regions. The size of the fast compartment was significantly lower in ARDS patients than in patients with healthy lung under all studied conditions.

**Conclusion:**

These results show that regional lung mechanics can be assessed by EIT. They reflect the lower respiratory system compliance of injured lungs and imply more pronounced regional recruitment and derecruitment in ARDS patients.

## Background

Acute respiratory distress syndrome (ARDS) is a severe inflammatory disease with a heterogeneous lung tissue damage affecting regional lung aeration, ventilation and perfusion and their spatial distribution. The resulting ventilation-perfusion mismatch is the most frequent cause of gas exchange impairment. The patients with ARDS require artificial ventilation which should secure adequate gas exchange and cause minimal harm to the already injured tissue. The selection of adequate tidal volume and positive end-expiratory pressure (PEEP) is crucial [1,2].

PEEP is used in patients with injured lungs to avoid cyclic recruitment and derecruitment and to homogenize the distribution of ventilation [3]. PEEP setting during artificial ventilation is a challenging procedure [4]. An inadequately high PEEP is harmful, since it results in overdistention of lung regions [5], while an inadequately low PEEP causes atelectasis. The knowledge of regional lung mechanics might provide useful information on the lung tissue response to modified ventilator settings.

The respiratory time constant (τ) characterizes the mechanical properties of the respiratory system [6]. τ is affected by PEEP [7-10]. The inflation and deflation behavior of the lung in ARDS patients can be described with a two-compartment model comprising lung tissue with a fast and a slow τ [7,11]. It is very likely that recruitment and derecruitment occur in the lung tissue exhibiting slow τ [11]. Reduced volume of atelectatic tissue, induced for instance by adequate PEEP choice, can be expected to reduce the amount of lung tissue with slow τ.

The assessment of regional τ is highly interesting in ARDS patients due to the inhomogeneous distribution of lung damage. The first measurement of regional respiratory τ was accomplished in an animal model of the acute respiratory distress syndrome (ARDS) using dynamic multiscan computed tomography (CT) [11]. However, this procedure of measuring regional τ is not feasible in the clinical setting because of the high radiation exposure, the danger associated with the patients' transport and high costs.

With the development of electrical impedance tomography (EIT) there is an imaging modality available that allows continuous non-invasive measurement of regional lung function without radiation at the bedside. The high scan rates of EIT allow the assessment of rapid changes in regional lung aeration [12-15].

The main objectives of our study were: 1) the assessment of regional τ by EIT in ARDS patients in comparison with lung healthy patients during step increase and decrease in airway pressure; 2) the evaluation of the PEEP effect under these conditions.

## Methods

### Subjects and examination protocol

The study protocol was approved by the institutional review board of the Medical Faculty of the Christian Albrechts University in Kiel, Germany. Written, informed consent to participate in the study was obtained from 32 patients or their legal representatives.

We included 12 lung healthy patients (36 ± 16 yr, mean age ± SD) with no history of smoking or lung disease and 20 patients with ARDS (58 ± 14 yr) with a mean ratio of arterial partial pressure of O_2_ to fraction of inspired O_2_ (PaO_2_/F_I_O_2_) of 164 ± 53 mm Hg. Mean PaO_2_ was 102 ± 17 mm Hg. ARDS patients were ventilated with a tidal volume of 7.2 ± 1.5 ml/kg body weight and a respiratory rate of 17 ± 5 breaths/min. Further details on the studied patients are shown in Table 1 and Table 2.

**Table 1:** Characteristics of patients with no lung disease

**Patient**	**Gender**	**Age, yr**	**BMI, kg/m**^**2**^	**PEEP**^**1**^**, cm H**_**2**_**O**	**Indication for surgery**
1	M	64	28	5	radical prostatectomy
2	M	38	27	5	cholecystectomy
3	M	57	24	5	fracture lower leg
4	M	19	28	5	arthroscopy
5	M	29	24	5	removal of osteosynthesis material
6	F	29	21	5	cholecystectomy
7	M	21	28	5	arthroscopy
8	F	20	24	5	cholecystectomy
9	M	26	26	5	fracture foot
10	M	36	24	5	testicular cancer
11	M	58	30	5	oral cancer
12	M	31	28	5	laparatomy, Crohn’s disease

BMI, body mass index; F, female; M, male; PEEP, positive end-expiratory pressure.

^1^PEEP during baseline conditions.

**Table 2:** Characteristics of patients with acute respiratory distress syndrome

**Patient**	**Gender**	**Age, yr**	**BMI, kg/m**^**2**^	**PEEP**^**1**^**, cm H**_**2**_**O**	**P**_**a**_**O**_**2**_**/F**_**1**_**O**_**2**_**, mmHg**	**Diagnosis**
1	F	69	25	7	200	pneumonia
2	M	54	19	15	138	pneumonia, sepsis
3	M	77	26	10	143	abdominal sepsis
4	F	57	24	10	233	polytrauma, massive transfusion
5	M	70	30	10	92	abdominal sepsis, pneumonia, MOF
6	M	66	24	15	128	pneumonia, sepsis
7	M	58	27	14	136	abdominal sepsis, cirrhosis of the liver
8	M	51	25	12	187	aspiration pneumonia, myocardial infarction
9	M	45	28	16	256	polytrauma
10	M	56	30	13	89	polytrauma, lung contusion
11	F	56	27	10	180	intracerebral bleeding, pneumonia
12	F	19	24	7	116	polytrauma, massive transfusion
13	F	54	26	8	210	pneumonia
14	M	63	26	10	204	abdominal sepsis, MOF
15	M	57	22	10	256	abdominal sepsis, MOF
16	F	50	28	10	144	abdominal sepsis, MOF
17	M	88	28	12	155	pneumonia
18	M	56	25	10	92	acute necrotic pancreatitis
19	M	55	25	10	200	polytrauma
20	M	52	35	10	119	aspiration pneumonia

BMI, body mass index; F, female; F_1_O_2_, fraction of inspired O_2_; M, male; MOF, multiple organ failure; PaO_2_ arterial pressure of O_2_; PEEP, positive end-expiratory pressure.^1^ PEEP during baseline condition.

All patients undergoing this study were sedated, paralyzed and artificially ventilated through an endotracheal tube (Mallinckrodt Medical, Athlone, Irland) with an inner diameter of 7.5/8.5 mm in female/male patients, respectively. Tidal volume of 6 ml/kg predicted body weight was used in all patients. In lung healthy patients, PEEP was set to 5 cm H_2_O whereas it was more than twice as high in the ARDS patients (11 ± 3 cm H_2_O, mean PEEP ± SD) (see Table 1 and Table 2). During the study examination, sustained stepwise increases in airway pressure from PEEP levels of 0, 8 and 15 cm H_2_O to the maximum pressure of 35 cm H_2_O were applied in apnea in random order. The high pressure was kept constant for one minute. Afterwards, patients exhaled passively against the PEEP valve with the pressure set to the initial PEEP value before the inflation. A recruitment maneuver with 35 cm H_2_O of PEEP applied for 45 s was performed in each patient after each inflation-deflation maneuver. Between the maneuvers, the patients were ventilated at the PEEP level of the next pressure step for ten minutes.

### EIT data acquisition and analysis

EIT examinations were performed with the Goe-MF II device (CareFusion, Höchberg, Germany). Sixteen electrodes (Red Dot 2239, 3M Health Care, Borken, Germany) were placed on the chest circumference in one transverse plane approximately at the level of the 5^th^ intercostal space. EIT data were acquired using an adjacent current (50 kHz, 5 mA_rms_) injection and adjacent voltage measurement protocol at a rate of 25 scans/s. A modified Sheffield back-projection image reconstruction procedure was used to generate the individual scans of impedance change (ΔZ) relative to a reference impedance distribution obtained during last four to five seconds in the plateau phase immediately preceding the step inflation from different PEEP levels. (Further details on EIT technology and image reconstruction can be found elsewhere, e.g. [16-18].)

The time behavior of regional ΔZ during inflation and deflation was analyzed under a priori assumption of a two-compartment behavior using a custom-made toolbox (MATLAB v.7, The Mathworks, Natick, USA). To analyze the regional respiratory time constants, the ΔZ waveforms in the ventral and dorsal lung areas within the studied chest cross-section were fitted using the bi-exponential equations of A = A_1_ ∙ (1 - e^-(t/τ)^_1_) + A_2_ ∙ (1 - e^-(t/τ)^_2_) for the inflation maneuver and A = A_1_ ∙ e^-(t/τ)^_1_ + A_2_ ∙ e^-(t/τ)^_2_ for the deflation maneuver. In these equations, A_1_ and A_2_ represent the regional fast and slow lung compartments which on inflation and deflation fill or empty with the fast or slow respiratory time constants τ_1_ and τ_2_. Finally, the fraction accounted for by the faster of the two components was calculated for each inflation and deflation at all three PEEP levels studied.

### Statistical analysis

The results in the text and figures are presented as mean values ± SD. Statistical analysis was performed using GraphPad Prism version 5.0 (GraphPad Software, San Diego, USA). The data were tested for normal distribution with the Kolmogorov-Smirnov normality test. The significance of differences between the patients with healthy lungs and the ARDS patients was tested using the Student′s unpaired t test. Student′s paired t test was applied to test the significance of differences between the values in the ventral and dorsal areas. One-way ANOVA for repeated measures with Bonferronipost test was used to assess the effect of PEEP. P values < 0.05 were considered significant and P values < 0.001 were considered highly significant.

## Results

**Figure 1:**
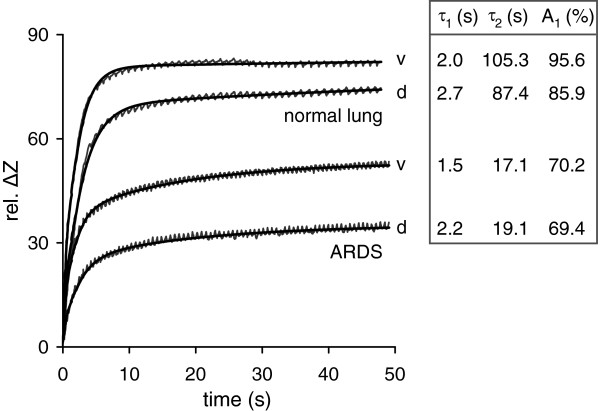
**Representative regional electrical impedance tomography (EIT) waveforms of relative impedance change (*rel. ΔZ*) obtained in a 57-year old male patient with normal lungs and a 52-year old male patient with acute respiratory distress syndrome (*ARDS*) during a step increase in airway pressure from 0 to 35 cm H**_**2**_**O.** Original data and data fitted to a bi-exponential equation in the ventral (*v*) and dorsal (*d*) chestregions are shown. The calculated regional fast (*τ*_*1*_) and slow respiratory time constants (*τ*_*2*_) and the relative size of the fast compartment (*A*_*1*_) are displayed to the right of each waveform. (The very small periodic fluctuations discernible in the original waveforms are synchronous with the heart beat and reflect the small changes in rel. ΔZ occurring at a rate of 59/min in the patient with normal lungs and of 92/min in the patient with ARDS).

EIT scanning was successfully accomplished during all studied conditions. Bi-exponential fitting of regional EIT waveforms in the ventral and dorsal lung regions was performed off-line with excellent goodness of fit. In the healthy subjects, R^2^ was in the range of 0.9914-0.9990 during the step increase and 0.9940-0.9991 during the step decrease in airway pressure. The corresponding values in the ARDS patients were 0.9689-0.9989 and 0.9707-0.9975. Representative regional EIT waveforms and the corresponding fitted curves obtained in one subject with normal lungs and in one ARDS patient during the step increase in airway pressure from 0 cm H_2_O to 35 cm H_2_O are shown in Figure 1. The ARDS patient exhibited lower values of regional τ_1_ and τ_2_ and the fast compartment was relatively smaller.

### Fast and slow respiratory time constants during step inflation

**Figure 2:**
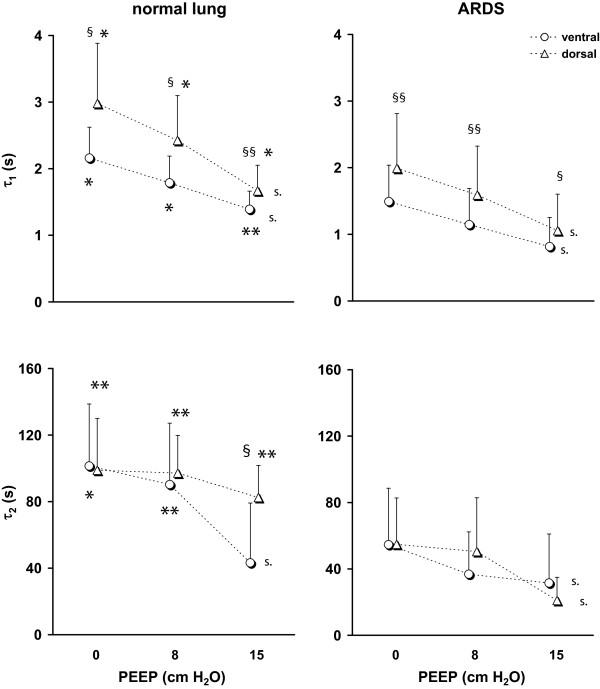
**Regional τ**_**1**_**(*top*) and τ**_**2**_**(*bottom*) determined by EIT in the ventral and dorsal lung areas in 12 patients with normal lungs (*left*) and 20 patients with ARDS (*right*) during step increase in airway pressure from 0, 8 and 15 cm H**_**2**_**O to the final pressure of 35 cm H**_**2**_**O.** Significant differences between and within the groups are indicated: normal lung versus ARDS *, p < 0.05, **, p < 0.001; ventral versus dorsal lung area §, p < 0.05, §§, p < 0.001; significant effect of positive end-expiratory pressure (*PEEP*), s., p < 0.05.

Regional respiratory time constants differed significantly between the patients with normal and injured lungs (Figure 2). ARDS patients exhibited significantly lower τ_1_ at all three studied PEEP values both in the ventral and dorsal regions. Regional τ_2_ was also lower in ARDS patients during all studied maneuvers. Only at the highest PEEP of 15 cm H_2_O, no significant difference in τ_2_ was found between the groups in the ventral region.

In both groups of patients, τ_1_ was significantly higher in the dorsal than in the ventral regions at all PEEP levels. Values of τ_2_ were similar in the ventral and dorsal regions in lung healthy patients as well as in ARDS patients. However, a pronounced decrease in τ_2_ in the ventral region was noted in patients with healthy lungs at the highest PEEP.

PEEP exerted a significant effect on regional τ_1_ and τ_2_. τ_1_ fell with PEEP in both patient groups. Regarding τ_2_, the effect of PEEP was mainly detected at the highest PEEP of 15 cm H_2_O and τ_2_ was not significantly affected by PEEP in the dorsal regions in the patients with normal lungs.

### Fast and slow respiratory time constants during step deflation

**Figure 3:**
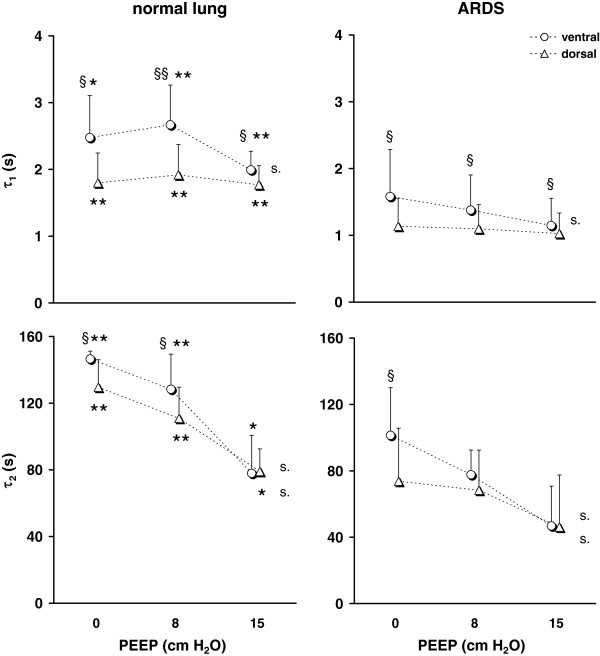
**Regional τ**_**1**_**(*top*) and τ**_**2**_**(*bottom*) determined by EIT in the ventral and dorsal lung areas in 12 patients with normal lungs (*left*) and 20 patients with ARDS (*right*) during passive deflation against the PEEP valve from the pressure of 35 cm H**_**2**_**O to the PEEP levels of 0, 8 and 15 cm H**_**2**_**O.** Significant differences between and within the groups are indicated: normal lung versus ARDS *, p< 0.05, **, p < 0.001; ventral versus dorsal lung area §, p < 0.05, §§ p < 0.001; significant effect of PEEP, s., p < 0.05.

τ_1_ and τ_2_ showed a significant difference between the lung healthy and the ARDS patients with the lower values found in the ARDS patients under all studied conditions (Figure 3).

A significant difference existed between τ_1_ in the ventral and dorsal lung areas under all studied conditions in both patient groups with lower values determined in the dorsal lung areas. τ_2_ was higher or tended to be higher in the ventral than in the dorsal lung areas when the passive exhalation ended at PEEP of 0 and 8 cm H_2_O whereas no regional differences were observed at 15 cm H_2_O of PEEP.

During step decrease in airway pressure, τ_1_ was only affected by PEEP in the ventral regions whereas no significant effect of PEEP was detected in the dorsal regions both in the patients with normal lungs and in the ARDS patients. τ_2_ was significantly influenced by PEEP in both lung regions whereby the values fell with increasing PEEP in both patient groups.

### Fast and slow lung compartments during step inflation

**Figure 4:**
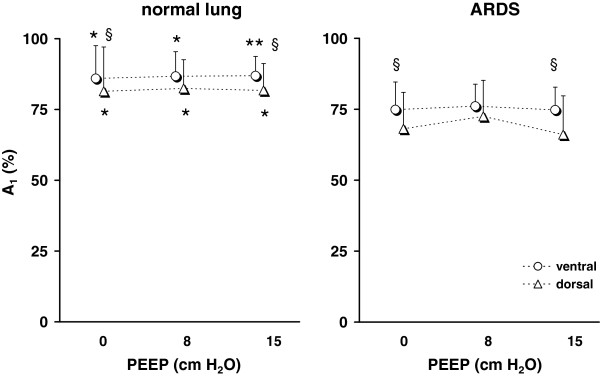
**Relative size of A**_**1**_**determined by EIT in the ventral and dorsal lung areas in 12 patients with normal lungs (*left*) and 20 patients with ARDS (*right*) during step increase in airway pressure from 0, 8 and 15 cm H**_**2**_**O to the final pressure of 35 cm H**_**2**_**O.** Significant differences between and within the groups are indicated: normal lung versus ARDS *, p < 0.05, **, p < 0.001; ventral versus dorsal lung area §, p < 0.05.

The compartment sizes were significantly different between the lung healthy and the ARDS patients under all three studied conditions. In the ARDS group, significantly lower fractions of the fast compartment were found (Figure 4).

The relative size of the fast compartment was lower in the dorsal than in the ventral lung areas. This finding was observed both in the patients with normal lungs and in the ARDS patients during step inflations from all PEEP values.

The fractions of the lung area exhibiting fast and slow filling were not significantly affected by different starting PEEP levels in either group of patients.

### Fast and slow lung compartments during step deflation

**Figure 5:**
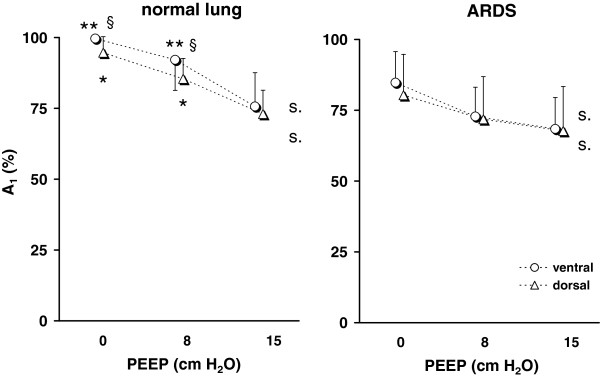
**Relative size of A**_**1**_**determined by EIT in the ventral and dorsal lung areas in 12 patients with normal lungs (*left*) and 20 patients with ARDS (*right*) during passive deflation against the PEEP valve from the pressure of 35 cm H**_**2**_**O to 0, 8 and 15 cm H**_**2**_**O, respectively.** Significant differences between and within the groups are indicated: normal lung versus ARDS *, p < 0.05, **, p < 0.001; ventral versus dorsal lung area §, p < 0.05; significant effect of PEEP, s., p < 0.05.

The fraction of the lung area in the studied chest cross-section accounting for the faster of the two compartments was significantly higher in the patients with normal lungs than in the ARDS patients when the deflation ended at a PEEP of 0 and 8 cm H_2_O (Figure 5). At 15 cm H_2_O, the differences between the groups were not significant. This means that, similar to the inflation maneuvers, the ARDS patients had a higher percentage of lung areas with τ_2_.

A significant difference between the ventral and the dorsal lung areas was only seen in the lung healthy patients during the deflation maneuvers ending at 0 and 8 cm H_2_O of PEEP. During these maneuvers, the fractions of the fast compartments were lower in the dorsal lung areas.

A significant effect of PEEP was seen in the lung healthy and in the ARDS patients during all maneuvers. The fractions of the fast compartment fell with increasing PEEP.

## Discussion

The results of our study indicate that EIT is able to assess the dynamic changes of regional lung aeration in response to step increase and decrease in airway pressure at the bedside. Regional lung inflation and deflation could be described using a two-compartment model. EIT-derived measures of regional respiratory mechanics τ_1_ and τ_2_ as well as the fractions of the fast and slow compartments clearly distinguished the ARDS patients from the patients with normal lungs. Regional differences in these measures were detected between the ventral and dorsal lung areas reflecting the different filling and emptying behavior of the lung tissue in the dependent and non-dependent regions. PEEP exerted a significant effect on regional τ_1_ and τ_2_ during both inflation and deflation. The fractional contribution of the fast and slow compartments depended on PEEP during step decrease but not during the step increase in airway pressure.

### ARDS vs. Normal lungs

EIT-based analysis of the temporal behavior of regional lung filling and emptying discriminated the ARDS patients from the patients with healthy lungs under all studied conditions. τ_1_ and τ_2_ were always higher in the patients with normal lungs than in the patients with ARDS most likely reflecting the higher lung compliance of the healthy subjects [19-21]. The only exception was τ_2_ in the ventral lung regions at the highest PEEP. In this case, regional τ_2_ fell markedly in the patients with normal lungs due to postulated overdistension and became similar to the value found in this region in ARDS patients.

Consistent with alveolar recruitment taking place in the injured lungs during step inflation and derecruitment during deflation is the finding of lower fractions of the fast compartment in the ARDS patients during both step increase and decrease in airway pressure. However, we observed that the fractional contribution of the two compartments to lung emptying was similar in ARDS patients and in patients with healthy lungs during expiration to the highest PEEP. The fractions of the slow compartment were higher in ARDS patients than in the patients with normal lungs at the lower PEEP values implying more rapid derecruitment in the injured lungs.

### Ventral vs. Dorsal regions

Regional differences between the EIT-derived measures of regional lung filling and emptying behavior τ_1_ and τ_2_ as well as between the relative sizes of the fast and slow compartments existed between the ventral and dorsal regions. These can be attributed to the effect of gravity. Derecruitment preferably occurred in the dependent lung regions during expiration and these regions became recruited again during inspiration, whereas overdistension was more likely present in the non-dependent lung areas [22].

Since regional alveolar distension is lower in the dependent than in the non-dependent lung regions at lung volumes below full inflation [23], a larger volume gain will occur in the dependent than in the non-dependent regions during sustained step inflation. This was found in our study as revealed by higher τ_1_ in the dorsal regions during inflation in both patient groups. The differences between the regions tended to be the largest when zero end-expiratory pressure was used. Regional τ_2_ values in the ventral and dorsal regions were alike during inflation at the lowest PEEP but fell more rapidly in the ventral regions. This phenomenon is attributable to regional lung overdistension which was observed at the PEEP of 15 cm H_2_O in the patients with healthy lungs and already at 8 cm H_2_O in the ARDS patients.

The effects of step decrease in airway pressure on regional dynamics of lung aeration can not be regarded as a simple reversal of the effects elicited by the rapid inflation of the lungs using a sustained step increase in airway pressure. Regional τ_1_ was lower in the dorsal than in the ventral regions during step deflation. τ_2_ was also lower in these regions when the deflation ended at the two lower PEEP values in the healthy lungs and at the lowest value in the injured lungs. τ_2_ was generally higher during deflation than inflation suggesting that once the lung was recruited during sustained inflation the regional loss in volume was slower than its gain during the step inflation.

Regional fraction of the fast compartment was higher in the ventral than in the dorsal regions. This can be attributed to more pronounced lung recruitment in the regions near the spine and has already been postulated in an experimental study using an animal model of ARDS [11]. The slow compartments were also larger in the dorsal regions during deflation to low PEEP values. This phenomenon may reflect regional derecruitment which was more probable the lower the PEEP value at which the deflation ended.

### PEEP effect

A PEEP-dependent fall in τ_1_ was found in both patient groups during step inflation as a result of decreasing respiratory system compliance and airway resistance at higher lung volumes [20,24,25]. The dependency of τ_1_ on PEEP also existed during deflation but it was only observed in the ventral regions. These results are in harmony with two clinical studies in patients with ARDS and chronic obstructive lung disease where a reduction of overall expiratory τ was found with increasing PEEP [10,26].

τ_2_ also exhibited a PEEP-dependent behavior with the lowest values found at the highest PEEP. This was expected because the lowest degree of recruitment or derecruitment occurred when the step inflation started and the step deflation ended at the highest PEEP of 15 cm H_2_O. High PEEP was shown to decrease expiratory resistance by preventing airway closure [26].

We expected that the slow compartment size would become smaller at higher PEEP during the inflation maneuver but this was not confirmed by the current results. The relative size of the slow compartment was previously shown to fall when lung tissue was recruited in an animal model of ARDS [11]. However, these results are not directly comparable with our findings obtained in patients using another imaging modality. In the present findings, the effect of recruitment on the slow compartment size may have been masked by other opposing effects like overdistension with increasing lung tissue resistance slowing down the filling of the regions with air. During the deflation maneuver, a marked dependency on PEEP was observed. The size of the fast compartment was the highest during lung emptying to 0 cm H_2_O of pressure, the corresponding average values exceeded 94% in the lung healthy subjects. This corresponds to previous findings showing an almost mono-exponential emptying pattern under these conditions [11].

### Limitations

The results of our study require cautious interpretation. The used set-up, the protocol, the imaging method and the assessment of regional and not global lung behavior had an impact on the obtained results and differed from previous studies on respiratory mechanics in ARDS.

The patient examinations were accomplished in a clinical setting in intubated, mechanically ventilated patients. Therefore, the findings reflect not only the dynamic behavior of the respiratory system alone but were also influenced by the endotracheal tube and the respirator circuit. Therefore, the τ_1_ and τ_2_ values were higher in our patients than in other studies [10,26-28].

The large pressure differences applied during the inflation and deflation maneuvers resulted in high flow rates. Smaller PEEP differences and, thus, lower flow rates were applied in a few previous studies [29,30]. Since the endotracheal tube is a flow-dependent resistive element it contributes to the determined τ along with the tubing and ventilator resistance affecting the analysis of the respiratory system mechanics [31]. High flows in the endotracheal tube and respirator circuitry increase the resistance and lead to higher respiratory τ.

During large airway pressure steps, additional phenomena may have influenced the EIT-derived findings: Firstly, pulmonary fluid and blood content may have been affected by the sustained inflation and deflation. This may have contributed to the small sloping plateau of the EIT waveforms in the patients with normal lungs during inflation and also to the steeper slope observed in the ARDS patients, where an additional effect of lung recruitment was probable. The relatively high τ_2_ values in a relatively small slow compartment found in the lung healthy patients are consistent with this effect. Secondly, continuing gas exchange during the later phases of the deflation maneuvers may have induced a prolonged decrease in ΔZ and contributed to the relatively high τ_2_ values. Thirdly, age-dependent differences in lung mechanics [32] may have impacted the results since the ARDS patients were older than the patients with healthy lungs. Fourthly, slightly different sections of the lung may have been examined by EIT at different PEEP steps because of a shift in the cranio-caudal axis. This phenomenon has been described in animal [33] and clinical EIT studies [34] but it is less dramatic compared when other imaging modalities are used since EIT examines a broader than a strictly two-dimensional section of the chest [35].

## Conclusions

EIT was able to describe regional lung filling and emptying behavior induced by step increase and decrease in airway pressure. Regional dynamics of lung aeration under these conditions could be characterized by regional fast and slow respiratory τ using a two-compartment model. These EIT-based measures were capable of identifying the differences in regional lung behavior between patients with ARDS and patients with normal lungs. These results are promising as they reflect clinically relevant phenomena like alveolar recruitment and derecruitment or overdistension. We expect that this EIT-based approach to analysis of pulmonary ventilation dynamics, possibly with a slightly modified protocol and setup to minimize the effect of some of the described study limitations, might be of benefit to optimize the respirator settings in the future.

## Competing interests

The authors declare that they have no competing interests.

## Authors’ contributions

SP, NW, IF have made substantial contributions to conception and design of the study. SP, MK, GE, DS, BV, NW, IF have made substantial contributions to acquisition of data, analysis and interpretation. All authors read and approved the final manuscript.

## Grants

The Medical Faculty of the Christian Albrechts University in Kiel, Germany, has supported BV as clinical researcher by funding a one-year rotational position.
